# Infectious Causes of Death: An Autopsy-Based Study of 546 Cases

**DOI:** 10.1093/ofid/ofaf065

**Published:** 2025-02-04

**Authors:** Soudabeh Eshaghi, Fereshte Sheybani, Aria Hedjazi, HamidReza Naderi, Matin Shirazinia, Negar Morovatdar

**Affiliations:** Department of Infectious Diseases and Tropical Medicine, Faculty of Medicine, Mashhad University of Medical Sciences, Mashhad, Iran; Department of Infectious Diseases and Tropical Medicine, Faculty of Medicine, Mashhad University of Medical Sciences, Mashhad, Iran; Legal Medicine Research Center, Iranian Legal Medicine Organization, Iran; Department of Infectious Diseases and Tropical Medicine, Faculty of Medicine, Mashhad University of Medical Sciences, Mashhad, Iran; Faculty of Medicine, Mashhad University of Medical Sciences, Mashhad, Iran; Clinical Research Development Unit, Imam Reza Hospital, Faculty of Medicine, Mashhad University of Medical Sciences, Mashhad, Iran

**Keywords:** autopsy, diagnostics errors, forensic medicine, infectious diseases, mortality

## Abstract

**Background:**

Diagnostic accuracy in cases of infectious diseases is crucial for appropriate patient management and public health interventions. This retrospective study aimed to evaluate the most common causes of death caused by infectious diseases and the rate of agreement between clinical diagnoses and autopsy findings in individuals diagnosed with infectious diseases in Mashhad, Iran.

**Methods:**

Autopsy reports from March 2009 to February 2018 were analyzed for patients diagnosed with infectious diseases. Demographic data, clinical diagnoses, and autopsy results were collected and compared. Discrepancies between clinical and autopsy diagnoses were assessed, and potential predictors of diagnostic discrepancy were examined.

**Results:**

Among 28 451 autopsied cases, 546 (1.9%) were diagnosed with infectious diseases. Pleuropulmonary infections were the most common cause of death (69.8%) as determined by autopsy, followed by bloodstream infections (14.1%) and intra-abdominal infections (10.0%). Discrepancies between clinical and autopsy diagnoses were identified in 22.4% of cases. Pleuropulmonary infections had the highest frequency of diagnostic discrepancies (29.1%), followed by central nervous system (CNS) infections (15.8%).

**Conclusions:**

This study underscores the importance of autopsy in identifying diagnostic discrepancies and improving clinical practice in cases of infectious diseases. They also highlight the need for ongoing efforts to enhance diagnostic capabilities, particularly in challenging cases such as pleuropulmonary and CNS infections, to reduce the burden of misdiagnosis and improve patient outcomes.

## Teaser

Among infectious disease-related deaths, autopsies revealed a 22.4% diagnostic discrepancy rate. Pleuropulmonary infections were the leading cause of death and had the highest discrepancies (29.1%), followed by central nervous system infections (15.8%), highlighting gaps in clinical diagnostics.

## Graphical Abstract

**graphical abstract:**
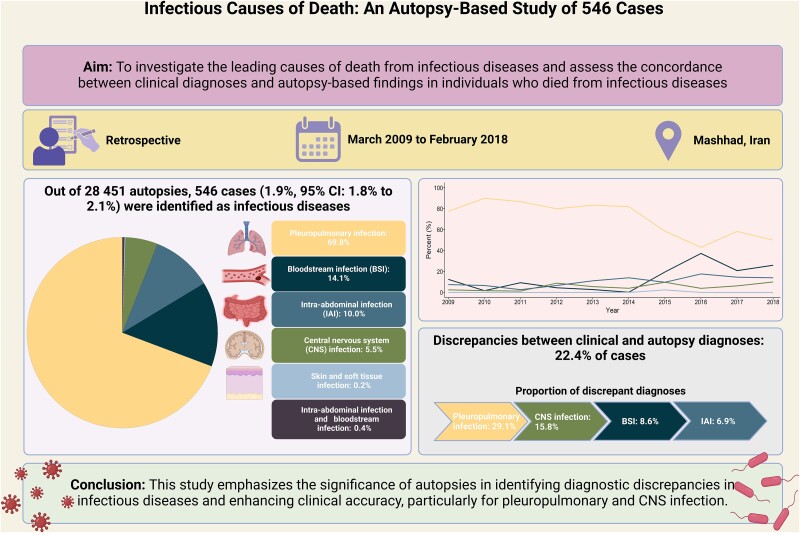
Created in BioRender. Mottahedi, M. (2025), https://BioRender.com/r19b720

Despite significant advancements in medical science, infectious diseases continue to be a major global health challenge, particularly in low-resource settings where their impact is most severe. The human and economic toll of communicable illnesses remains staggering, with an estimated economic burden reaching up to US$8 trillion for 8 major infectious diseases alone in 2016. These diseases, including human immunodeficiency virus (HIV)/AIDS, malaria, and tuberculosis (TB), collectively contribute to >156 million lost life years annually [1]. In 2019, mortality from infectious syndromes rivaled that of ischemic heart disease (9 million deaths) and neoplasms (10 million deaths), underscoring the immense toll infections exact as both underlying and intermediate causes of death [2]. Consequently, effective control and prevention strategies for infectious diseases are paramount in public health policy-making worldwide.

Although infectious syndromes contribute substantially to global mortality, data on this subject are scarce [2]. Conditions like HIV/AIDS [3], TB [4], malaria [5], and lower respiratory infections [6] exert considerable influence on mortality and morbidity, particularly in regions with limited access to healthcare services and preventive measures. Beyond these specific diseases, the emergence of novel infectious agents, exemplified by the coronavirus disease 2019 pandemic [7], underscores the persistent threat posed by infectious diseases. The rapid dissemination of new pathogens underscores the necessity of robust surveillance systems, effective public health interventions, and international collaboration to combat infectious diseases. Moreover, infections participate in the cause-of-death chain of communicable, maternal, neonatal, and nutritional diseases, noncommunicable diseases (NCDs), and injuries [2]. In particular, the coexistence of infectious diseases and NCDs presents complex challenges for global health systems. Individuals living with NCDs may be more susceptible to severe outcomes from infectious diseases, highlighting the interconnectedness of these health issues. Moreover, infectious diseases can exacerbate existing NCDs or lead to secondary complications, further amplifying the burden on healthcare systems and population health [8].

However, diagnosing infectious diseases accurately and promptly remains a persistent challenge [9], particularly in resource-limited settings, where diagnostic errors and delays can lead to harmful consequences, including inappropriate treatment and poor outcomes [9–11]. Autopsies have historically been pivotal in uncovering diagnostic discrepancies, especially in infectious diseases, by identifying subtle or unexpected findings that might be overlooked during clinical assessments [12–15]. While advancements in diagnostic technology—such as improved imaging and more sensitive laboratory methods—have significantly enhanced the detection of occult infections and other pathological processes, autopsies remain invaluable when imaging and laboratory findings are inconclusive or ambiguous [16]. These methods have improved diagnostic sensitivity and specificity but still leave critical gaps that autopsies uniquely address [16].

A 2005 systematic review revealed that the overall rate of disparity between clinical and autopsy-based diagnoses has remained largely unchanged over several decades, with autopsies uncovering previously unsuspected findings in up to 50% of cases [12]. Although autopsies do not directly impact the care of deceased patients, they provide insights that improve future patient management, validate diagnostic algorithms, and support outbreak control efforts. As infections, including those among immunosuppressed patients, become increasingly complex, autopsies reveal critical, previously undetected infections that highlight gaps in existing diagnostic methods. In this study, we detail the autopsy-based diagnoses, regarded as the gold standard, of individuals who succumbed to infectious diseases in Mashhad, Iran. Additionally, we examined the frequency of diagnostic discrepancies, which refer to differences between the clinical diagnoses made by attending physicians and the autopsy findings.

## METHODS

### Study Design

This study employed a retrospective analysis of autopsy reports conducted on individuals diagnosed with infectious diseases in Mashhad, Iran.

### Population

Mashhad is the capital of Razavi Khorasan province and the country's second-largest city, situated in the northeast Iran [17]. The study population included individuals whose autopsies were conducted by the Legal Medicine Organization (LMO) in Mashhad. As the LMO serves as a referral center, the cases are not limited to Mashhad residents and may include individuals from other cities and provinces.

### Data Collection

Autopsy reports from March 2009 to February 2018 were obtained from the LMO in Mashhad, Iran, a government entity responsible for forensic medicine and pathology services, including autopsies and determining causes of death. A structured checklist was used to extract demographic data (age, sex, and comorbidities), initial clinical diagnoses provided by attending physicians, and autopsy-based diagnoses. For patients admitted to the hospital prior to death, all medical records from the admission period were reviewed, ensuring that the clinical data reflected the most current health status of the patients leading up to their death. These records provided clinical diagnoses, treatments, and relevant medical histories. Furthermore, any missing data were completed using notes from the forensic setting, ensuring a comprehensive clinical profile for each case.

The study compared the primary cause of death based on clinical diagnoses with autopsy findings, the latter considered the gold standard. Cases where autopsy findings contradicted clinical diagnoses were classified as diagnostic discrepancies. Specifically, a discrepancy was defined as a case where the primary cause of death identified by autopsy differed from the attending physician's documented diagnosis. If an infection was noted in the medical records but not listed as the primary cause of death, it was classified as discrepant only if the autopsy identified the infection as the principal cause and the physician did not manage the patient based on the diagnosis of infection. Diagnostic discrepancies were only analyzed in cases where the deceased had been admitted to a hospital prior to death and their clinical documentation was complete.

In our study, infections were categorized as either community-acquired or healthcare-associated based on specific criteria. Community-acquired infections were defined as infections present or manifesting within 48 hours of hospital admission. These infections were generally not related to any recent hospital stay or medical intervention and included infections that were present before the patient's hospital admission. In contrast, healthcare-associated infections were defined as infections occurring ≥48 hours after hospital admission or infections that developed after a recent discharge from a hospital. This classification includes infections related to surgical procedures performed during the hospital stay, such as surgical site infections, as well as infections associated with prosthetic devices implanted during the hospital stay or shortly thereafter. Infections related to prosthetic devices implanted in the community and manifesting after a significant period following the implantation, without recent hospital exposure, were classified as community-acquired.

### Inclusion Criteria

The study included all cases in which autopsies performed between March 2009 and February 2018 identified an infectious disease as the primary cause of death. Both hospital-admitted patients and individuals who died outside the healthcare setting were included.

### Exclusion Criteria

For the analysis of diagnostic discrepancies, only cases with adequate medical documentation from hospital admissions prior to death were included. Patients without sufficient clinical documentation were excluded.

In Iran, autopsies are performed by forensic pathologists affiliated with the LMO, which investigates deaths under various circumstances, including suspicious or unexpected deaths, homicides, suicides, accidents, occupational hazards, and unclear causes. Autopsies are also conducted for maternal deaths, deaths within 24 hours of hospitalization with unknown causes, and upon family or physician requests. Forensic pathologists perform thorough postmortem examinations, analyzing internal organs, tissues, and fluids, as well as conducting toxicological, histopathological, and microbiological assessments, to determine the cause and manner of death. They also collaborate with law enforcement and medical professionals, providing expert opinions and testimony in legal contexts.

### Statistical Analysis

Continuous data were described with median and interquartile range (IQR) and categorical variables with frequency and percentage. To assess the relationship between different patient characteristics and the prevalence of discrepant diagnoses, we reported the prevalence ratio (PR) with the corresponding 95% confidence interval (CI). Poisson regression with robust variance was used to calculate the PR [18, 19]. To evaluate the agreement between the attending physician's diagnosis and the autopsy-based diagnosis, we reported the kappa coefficient using a weighted kappa, assigning a weight of 1.00 for complete agreement and a weight of 0 for all other cases. The threshold for statistical significance was set at <.05 for all analyses. All statistical analyses were done using R version 4.3.1 (R Development Core Team, University of Auckland, New Zealand) and Stata version 14.2 (Stata, College Station, Texas) software.

### Research Ethics

The ethics committee of Mashhad University of Medical Sciences approved this study with the code of IR.MUMS.MEDICAL.REC.1398.799. Due to the nature of this study and the preserved anonymity of patients, a waiver of informed consent was obtained by the ethics committee of Mashhad University of Medical Sciences. The study was conducted following the principles outlined in the Declaration of Helsinki.

## RESULTS

From March 2009 and February 2018, 28 451 autopsied cases were identified, of which 546 (1.9% [95% CI, 1.8%–2.1%]) were diagnosed with infectious diseases based on autopsy findings. Cases included 40 neonates (7.3%), defined as infants from birth to 28 days of age, and 108 elderly individuals (19.8%), defined as those aged ≥65 years (Table 1). The median length of hospital stay was 1 day (IQR, 1–2 days), with 409 individuals (74.9%) dying within 24 hours of admission.

**Table 1.** Clinical Characteristics and Autopsy-Based Diagnoses

Characteristic	Total (N = 546)
Age, y, median (IQR)	40 (25–60)
Neonates (≤28 d)	40 (7.3)
Elderly (≥65 y)	108 (19.8)
Sex (male)	405/545 (74.3)
Nationality	
Iranian	533 (97.6)
Iraqi	7 (1.2)
Pakistani	2 (0.4)
Turkmenistani	2 (0.4)
Afghan	1 (0.2)
Tajikistani	1 (0.2)
Referral center	
Teaching hospitals	450 (82.4)
Nonteaching hospitals	44 (8.1)
Police station	52 (9.5)
Underlying conditions	
Intravenous drug use	62/445 (13.9)
Immunocompromised	32/445 (7.2)
Diabetes mellitus	27/445 (6.1)
Hypertension	23/445 (5.2)
Ischemic heart disease	19/445 (4.3)
HIV/AIDS	20/445 (4.5)
Cancer	11/445 (2.5)
Pregnant	7 (1.6)
Autopsy-based diagnosis	
Pleuropulmonary infection	381 (69.8)
BSI	77 (14.1)
IAI	55 (10.0)
CNS infection	30 (5.5)
Skin and soft tissue infection	1 (0.2)
IAI and BSI	2 (0.4)
Discrepant diagnosis	70/312 (22.4)
Cases that changed from a noninfectious clinical diagnosis to an infectious autopsy-based diagnosis	21/70 (30.0)
Cases revised due to incorrect infectious diagnoses	49/70 (70.0)
LOS, d, median (IQR)	1 (1–2)
ICU admission	29 (5.3)
Mechanical ventilation	34 (6.2)
GIB	6 (1.1)

Data are presented as No. (%) unless otherwise indicated.

Abbreviations: BSI, bloodstream infection; CNS, central nervous system; GIB, gastrointestinal bleeding; HIV, human immunodeficiency virus; IAI, intra-abdominal infection; ICU, intensive care unit; IQR, interquartile range; LOS, length of hospital stay.

**Figure 1.**
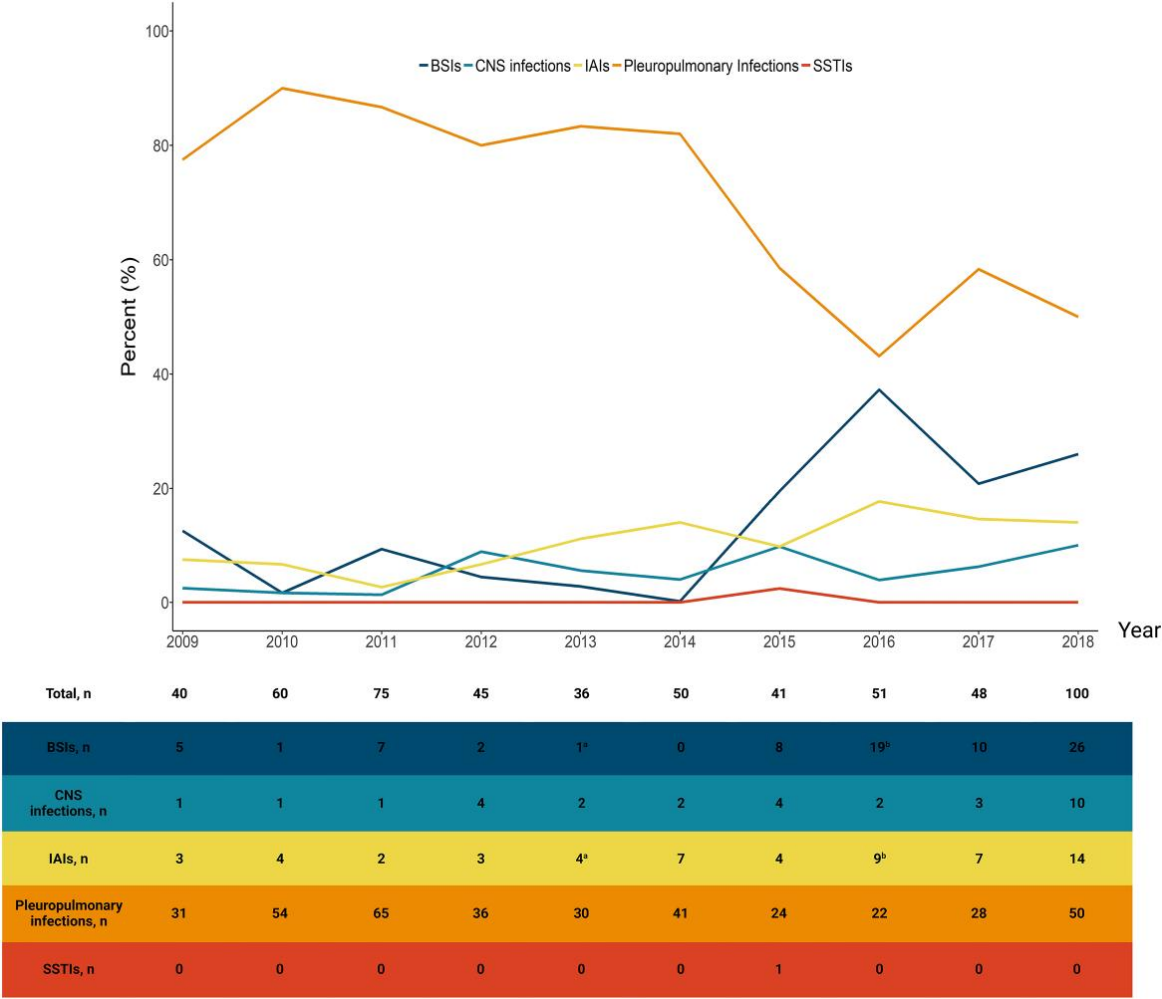
Annual frequency and absolute number of infection-related causes of mortality during the study period. ^a,b^ In each of the mentioned years, 1 patient had intra-abdominal infection and bloodstream infection as co-primary causes of death. Created in BioRender. Mottahedi, M. (2025), https://BioRender.com/a53t911. Abbreviations: BSIs, bloodstream infections; CNS, central nervous system; IAIs, intra-abdominal infections; SSTIs, skin and soft tissue infections.

The underlying conditions in adults included injection drug use in 62 of 445 (13.9%) cases, immunocompromised states in 32 (7.2%), diabetes mellitus in 27 (6.1%), hypertension in 23 (5.2%), HIV/AIDS in 20 (4.5%), ischemic heart disease in 19 (4.3%), cancer in 11 (2.5%), and pregnancy in 7 (1.6%). In children, the underlying conditions included preterm birth in 6 (6.1%); cerebral palsy in 3 (3.0%); and microcephalus, hydrocephalus, Down syndrome, esophageal atresia, encephalocele, cleft palate, and chronic kidney disease in 1 each. At postmortem examination, pleuropulmonary infections were the most common causes of death due to infectious diseases in 381 (69.8%), followed by bloodstream infections (BSIs) in 77 (14.1%), intra-abdominal infections in 55 (10.0%), central nervous system (CNS) infections in 30 (5.5%), and skin and soft tissue infections in 1 case (Table 1). Four hundred eighty-four (88.6%) cases died due to community-acquired infections and 62 (11.4%) due to healthcare-associated infections. Over the past decade, pleuropulmonary infections remained the leading infectious cause of death, despite showing a declining trend. A modest upward trend was observed in BSIs, while other causes remained stable (Figure 1).

**Figure 2.**
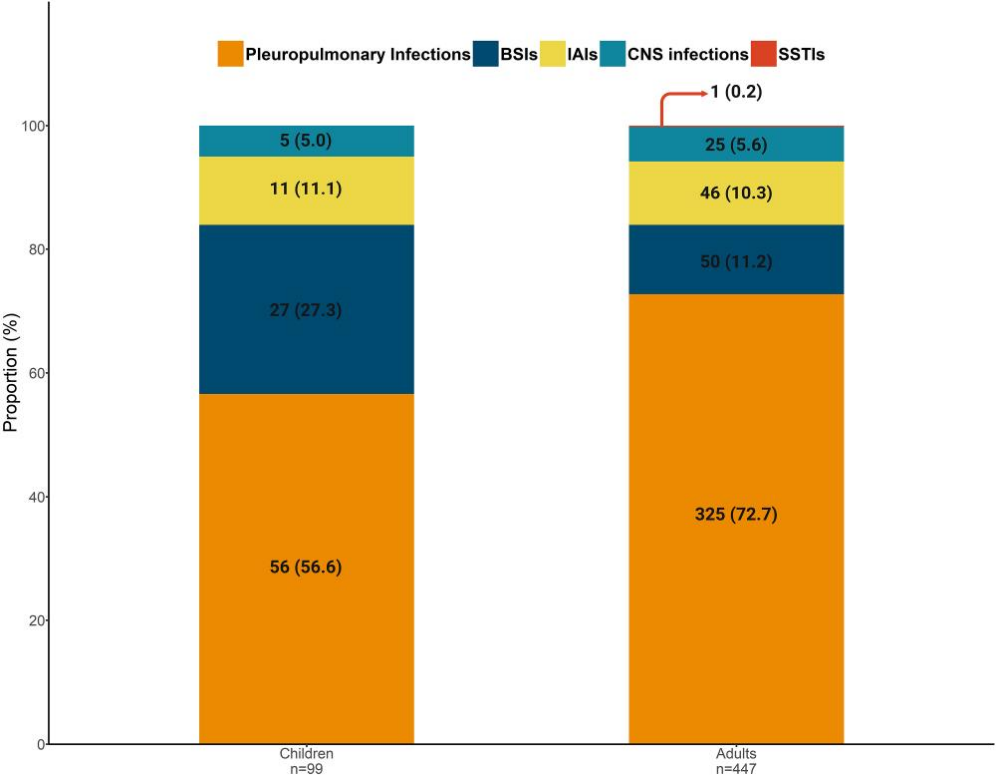
The proportion of infection-related causes of mortality among adults and children. Children are defined as individuals <15 years of age. Created in BioRender. Mottahedi, M. (2025), https://BioRender.com/j12c907. Abbreviations: BSIs, bloodstream infections; CNS, central nervous system; IAIs, intra-abdominal infections; SSTIs, skin and soft tissue infections.

Tuberculosis was identified as a major infectious cause of death in 46 (8.4%) cases. Among these, 42 cases (91.3%) were diagnosed with pulmonary TB, while the remaining 4 cases (8.7%) were classified as extrapulmonary TB, specifically TB meningitis. Moreover, among patients with HIV/AIDS, 5 (20.0%) had pulmonary TB. Pleuropulmonary infections, which include both pulmonary TB and other lung infections, were the leading cause of death as determined by autopsy in both age groups: 56 (56.6%) in children and 325 (72.7%) in adults (Figure 2).

The frequency of presumed diagnoses by the attending physician is shown in Table 2. Of the 312 cases for which clinical diagnoses were made by attending physicians before death, 70 (22.4%) diagnostic discrepancies were identified. It is important to note that the remaining cases included in the study were referred to forensic pathologists without clinical diagnoses, either from the police office or because the individuals were found deceased, and thus were not included in this subset. The frequency of discrepant diagnoses among those with pleuropulmonary infections as determined by autopsy was 60 of 206 (29.1%) cases, BSIs in 5 of 58 (8.6%) cases, intra-abdominal infections in 2 of 29 (6.9%) cases, and CNS infections in 3 of 19 (15.8%) cases. The frequency of diagnostic discrepancies among children and adults was 10 of 56 (17.9%) cases and 60 of 256 (23.4%) cases, respectively. The kappa coefficient between the attending physician's diagnosis and the autopsy-based diagnosis was 0.64 (95% CI, .57–.71).

**Table 2.** Clinical Diagnoses or Syndromes Presumed by Attending Physicians

Diagnosis	No. of Cases
Respiratory syndromes	
Pneumonia	89
Aspiration pneumonia	20
Pulmonary TB	22
ARDS	13
COPD exacerbation	3
Lung abscess	1
Sepsis/severe sepsis/septic shock	88
Peritonitis	26
Poisoning/intoxication/overdose	7
CNS infections	
Meningitis/meningoencephalitis	5
TB meningitis	4
Brain abscess	2
CNS infection	2
Gastroenteritis	5
Complications of multiple trauma	4
Encephalopathy	7
Myocardial infarction	2
Cardiogenic shock	2
Congestive heart failure	2
GIB, SSTI, PTE, preeclampsia, limb ischemia, septic emboli, peritoneal TB, cholangitis	1 each

Abbreviations: ARDS, acute respiratory distress syndrome; CNS, central nervous system; COPD, chronic obstructive pulmonary disease; GIB, gastrointestinal bleeding; PTE, pulmonary thromboembolism; SSTI, skin and soft tissue infection; TB, tuberculosis.

None of the binary characteristics investigated in this study, including age group (adults vs children: PR, 1.31 [95% CI, .72–2.40]; *P* = .378), sex (male vs female: PR, 1.00 [95% CI, .63–1.56]; *P* = .984), referral center (nonacademic vs academic: PR, 0.88 [95% CI, .39–1.99]; *P* = .765), and immune system status (immunocompromised vs immunocompetent: PR, 0.62 [95% CI, .24–1.56]; *P* = .307), were significantly associated with diagnostic discrepancies (Table 3).

**Table 3.** Comparing the Frequency of Different Variables Between Groups With Correct Diagnoses and Discrepant Diagnoses Using Univariable Poisson Regression

Characteristic	Variable	Discrepant Diagnoses (n = 70), No. (%)	Prevalence Ratio (95% CI)	*P* Value
Age group	Adult^a^ Children	60/256 (23.4) 10/56 (17.9)	1.31 (.72–2.40)	.378
Sex	Male Female	49/218 (22.5) 21 (22.6)	1.00 (.63–1.56)	.984
Referral center	Nonacademic center Academic center	5/25 (20.0) 65/287 (22.7)	0.88 (.39–1.99)	.765
Immunity status	Immunocompromised Immunocompetent	4/28 (14.3) 66/284 (23.2)	0.62 (.24–1.56)	.307

Abbreviation: CI, confidence interval.

^a^Adults are defined as individuals aged ≥15 years.

## DISCUSSION

Our study examined 28 451 autopsy cases over a decade, identifying 546 (1.9%) cases diagnosed with infectious diseases. Notably, neonates and the elderly constituted a substantial portion of these cases, comprising 7.3% and 19.8%, respectively. Pleuropulmonary infections were the leading cause of death, as determined by autopsy, followed by BSIs, intra-abdominal infections, CNS infections, and skin and soft tissue infections. In line with findings from our study, a systematic analysis in 2019 showed that respiratory infections and BSIs stand out as the most fatal infectious diseases globally. However, notable regional disparities exist that are particularly evident in the mortality rates associated with BSIs [2]. The true impact of BSIs may be underestimated due to the frequent attribution of deaths to the primary source of infection, such as pneumonia or urinary tract infections, rather than to the BSI itself [20]. In our study, pleuropulmonary infections were the leading cause of death in both adults and children, while BSIs, though less common, had a significant impact, particularly among children. This may be attributed to the high proportion of neonates in our study population, who are more vulnerable to sepsis [21, 22]. These findings highlight the need for targeted interventions for BSIs in pediatric populations, alongside continued efforts to combat pleuropulmonary infections across all age groups.

Our study uncovered a concerning rate of diagnostic discrepancies in cases of autopsied patients who died from infectious diseases, with 1 in 5 cases being misdiagnosed. This highlights a significant gap in accurate diagnosis. Autopsy has long been regarded as a critical tool for determining the true cause of death due to its ability to identify conditions that were otherwise undiagnosed or misdiagnosed during clinical care. While advances in modern imaging techniques have greatly improved diagnostic capabilities, they are not infallible, and autopsy remains an essential complement to these methods. This dual approach is supported by prior research, which highlights the continued value of autopsy in supplementing radiological findings to refine diagnoses and improve our understanding of disease processes [23]. Earlier studies have documented varying rates of diagnostic discrepancies identified through autopsy evaluations [24–31], emphasizing its utility across different age groups and healthcare settings, including public, private, and university-affiliated hospitals [32, 33].

We acknowledge that diagnosing infectious diseases is a complex process influenced by factors such as clinical judgment, diagnostic methods, and resource availability. Variations in clinical diagnostic methods, including the criteria applied and the tools available, can contribute to discrepancies between clinical and autopsy findings. In our study, the most common diagnostic discrepancies were identified in patients who died from pleuropulmonary and CNS infections. The clinical significance of these discrepancies could be notable, particularly because, in several cases, autopsy findings significantly altered the understanding of the cause of death compared to the initial clinical diagnosis. Similar issues were highlighted in a survey of physicians, which identified common errors in diagnosing infectious diseases, particularly upper respiratory tract infections, TB, pleuropulmonary infections, and CNS infections, often occurring during hypothesis generation and history-taking [11]. Inadequate patient history-taking, limited laboratory testing, and the misinterpretation of overlapping symptoms often complicate the diagnostic process, leading to misdiagnoses. These findings highlight the need for improved diagnostic processes that address these confounding factors.

Of particular concern in our study is the high frequency of diagnostic discrepancies observed in cases of pleuropulmonary infections, with nearly one-third of cases misdiagnosed, making them 3 times more likely to be misdiagnosed than other infection-related deaths. Commonly misdiagnosed or underdiagnosed conditions included pneumonia, pulmonary TB, and other respiratory infections. The diagnostic challenge arises from overlapping symptoms with noninfectious conditions like chronic obstructive pulmonary disease and heart failure, as well as the diverse etiology of pleuropulmonary infections. Previous autopsy studies have demonstrated the value of histological examination in revealing unsuspected pathogens and influencing clinical guidelines for pneumonia diagnosis and treatment [34]. Histological examination of postmortem lung tissue enhances diagnostic sensitivity by identifying causative pathogens, confirming antemortem microbiological findings, and assessing disease patterns, thereby aiding in the recognition of both infectious and noninfectious conditions, such as pneumonia mimics [34]. In line with the result of our study, a physician survey conducted in 2023 examining 465 cases of diagnostic errors regarding infectious diseases also found that upper respiratory tract infections, TB, and pleuropulmonary infections were most commonly misdiagnosed, supporting our findings [11].

The rapid progression to death within 24 hours of hospital admission in many pleuropulmonary infection cases highlights critical challenges in managing severely ill patients. While early mortality highlights the potential role of delayed presentation to healthcare facilities, diagnostic limitations, or delayed hospital admission, it also reflects the complexities of evaluating and treating patients in critical condition. Many such patients likely received empiric antibiotic therapy upon arrival, even if infection was not initially listed as a cause of death, indicating that clinicians may have recognized the possibility of infection and initiated treatment promptly. However, the abbreviated admission period often limits the ability to perform comprehensive diagnostic evaluations, such as blood cultures, imaging studies, or other tests, which are critical for definitive diagnosis and targeted treatment. Therefore, the diagnostic discrepancy rate of approximately 22% should not necessarily be interpreted as evidence of clinician oversight but may instead highlight systemic issues that should be studied further to ensure testing is done rapidly in critically ill patients. These findings emphasize the need to enhance systems for rapid evaluation and diagnosis of critically ill patients with suspected pleuropulmonary infections. Addressing barriers to timely healthcare access, improving diagnostic capabilities, and streamlining processes for early intervention are essential to mitigate the impact of these infections and reduce associated mortality rates. Furthermore, the requirement for autopsy in cases of death of unknown cause within the first 24 hours of presentation to healthcare centers in Iran could partially explain the high observed mortality rate within this timeframe.

Similarly, CNS infections exhibited a notable proportion of discrepant diagnoses, with approximately 16% of cases showing discrepancies. Infections of the CNS pose diagnostic challenges due to their variable clinical manifestations and the limitations of available diagnostic tests. We previously reported a high incidence of diagnostic errors in CNS infections, with 76.3% of cases admitted to the hospital involving such errors [35]. The low proportion of CNS infections in the current study may be partly explained by the lower frequency of brain autopsies, which are performed less often due to the need for special consent, technical and ethical considerations, and the complexity involved in such procedures. Previous research identified several contributors to diagnostic inaccuracies in patients with CNS infections, including failures in ordering appropriate tests, inadequate history-taking, missed epidemiological clues, incomplete clinical examinations, and misinterpretation of diagnostic tests. Significant associations were found between poor outcomes and delayed diagnosis and inappropriate empirical antibiotic therapy [35]. These findings underscore the importance of thorough evaluation and appropriate diagnostic procedures to minimize errors and improve management in cases of suspected CNS infections.

Our study found that HIV infection contributed to 4.5% of infectious disease mortality, a significant proportion compared to the general population's HIV prevalence of approximately 0.007% in Iran [36], highlighting the disproportionate impact of HIV/AIDS on mortality. However, this discrepancy may also reflect underreporting of HIV in the general population, emphasizing the need for targeted interventions and improved management strategies for HIV-positive individuals. Additionally, TB accounted for 8.4% of deaths from infectious diseases, with pulmonary TB being the predominant form (91.3% of TB-related deaths). The delayed diagnosis of TB can lead to increased transmission, higher medical costs, and greater mortality [37]. Several autopsy-based studies from around the world have shown that TB is often missed during the individual's lifetime and only diagnosed posthumously. These findings underscore the critical need for improved diagnostic protocols and early detection to reduce TB's impact on both individuals and public health [37].

The findings of our study reveal that a relatively low percentage of autopsy-studied deaths were attributed to infectious diseases. This contrasts with the higher prevalence of infectious diseases reported in non-autopsy-proven investigations [38–40]. Notably, the 10 leading causes of total years of life lost in 2016 included lower respiratory infections, diarrheal diseases, malaria, and HIV/AIDS, which are all communicable diseases with significant mortality impacts globally [41]. Several factors may contribute to this discrepancy. First, it is important to clarify that the infections identified through autopsy in our study were consistently the primary cause of death. We did not include cases where infections were secondary or contributory causes. Second, the low rate of autopsies conducted in cases where infectious complications are suspected plays a significant role. This phenomenon can be attributed to the specific regulatory circumstances in our country, which stipulate that autopsies are mandatory only under certain conditions, excluding deaths specifically resulting from infectious diseases. Consequently, a considerable number of deaths attributed to infectious diseases do not undergo autopsy procedures, resulting in their underrepresentation in our study.

Our study had several limitations. First, the potential for selection bias exists due to the specific criteria for performing autopsies in Iran, which may result in certain cases being more likely to undergo autopsy and skew the study sample. Additionally, our study focused on infectious syndromes and did not report cause-specific diagnoses, except for TB and HIV infection, limiting the depth of understanding regarding other infectious disease causes of death. Another limitation is the use of 15 years as the threshold for adulthood in Iran during the study period, which differs from the 18-year threshold used in many other countries. This discrepancy may introduce bias when comparing our findings with studies from countries with a higher age threshold. However, this was the standard at the time of data collection, reflecting the clinical and societal norms of that period. Furthermore, the high rate of diagnostic discrepancies in our study may be attributed to the rapid progression to death within 24 hours in many cases, which limited the time for comprehensive diagnostic evaluations and highlighted systemic challenges in diagnosing critically ill patients, rather than indicating errors in diagnosis. Despite these limitations, our study utilized a large dataset over a decade, providing a robust basis for analysis and offering valuable insights into the discrepancies between clinical diagnoses and autopsy findings in infectious diseases.

## CONCLUSIONS

Our study underscores the critical role of autopsy in identifying diagnostic discrepancies and improving clinical accuracy, particularly in challenging cases such as pleuropulmonary and CNS infections. It highlights the need for continuous efforts to enhance diagnostic capabilities, improve healthcare access, and optimize preventive measures like vaccination to reduce the burden of misdiagnosis and infectious diseases burden. Future research should explore these challenges in greater detail, potentially through qualitative studies or structured interviews with healthcare providers. Accurate diagnosis is essential for effective patient management and improved outcomes, emphasizing the necessity for ongoing training, enhanced access to diagnostic resources, and systematic approaches to clinical assessments.

## Notes

***Author contributions.*** S. E.: Formal analysis, investigation, writing–original draft. F. S.: Conceptualization, supervision, investigation, methodology, writing–review & editing. A. H.: Conceptualization, methodology, project administration. H. R. N.: Conceptualization, methodology, project administration, supervision, writing–review & editing. M. S.: Formal analysis, methodology, writing–review & editing. N. M.: Formal analysis, investigation, writing–original draft.

***Acknowledgments.*** We would like to thank Dr Mehran Mottahedi for assisting us in generating our figures. Part of our figures were created using BioRender (https://BioRender.com).

***Financial support.*** This research was conducted with funding support from the vice-chancellery for research of Mashhad University of Medical Sciences (research project number 980905 and ethic code of IR.MUMS.MEDICAL.REC.1398.799) and is part of the thesis of the first author.
